# Insight into the antifungals used to address human infection due to *Trichosporon* spp.: a scoping review

**DOI:** 10.2217/fmb-2021-0048

**Published:** 2021-10-25

**Authors:** Amanda M Malacrida, Tânia P Salci, Melyssa Negri, Terezinha IE Svidzinski

**Affiliations:** ^1^Departament of Clinical Analyses and Biomedicine, Universidade Estadual de Maringá, Maringá, Paraná, CEP, 87020-900, Brazil; ^2^Departament of Pharmacy and Science, Faculdade Integrado de Campo Mourão, Campo Mourão, Paraná, CEP, 87300-970, Brazil

**Keywords:** experimental assays, potential drugs, susceptibility test, treatment, trichosporonosis

## Abstract

Trichosporonosis infections have been increasing worldwide. Providing adequate treatment for these infections remains a challenge. This scoping review contains information about potential antifungals to treat this pathology. Using online databases, we found 76 articles published between 2010 and 2020 related to this topic. Classic antifungals, molecules and biomolecules, repositioned drugs and natural products have been tested against species of *Trichosporon*. Experimental research has lacked depth or was limited to *in vitro* and *in vivo* tests, so there are no promising new candidates for the clinical treatment of patients with trichosporonosis. Furthermore, most studies did not present appropriate scientific criteria for drug tests, compromising their quality.

Species of *Trichosporon* have emerged as a common human pathogen over the past four decades. Invasive trichosporonosis is the second most common noncandidal fungal infection [1–4]. Mortality rates from this infection vary (from 30 to 90%) and are directly related to the immunological and general conditions of the patient [5–8]. However, there is no adequate pharmacotherapy for the treatment of this pathology because different clinical isolates of the same species have great variability in antifungal susceptibility tests [9–11].

*Trichosporon* spp. are basidiomycetes with dimorphic characteristics. It can be easily found in the environment (soil, water and animals) and in human microbiota (gastrointestinal tract, skin and upper respiratory tract) [1,4,7,12]. In the past, this fungal genus comprised 50 species [1]. However, the most recent taxonomic study based on the sequencing IGS1 rDNA identified 20 species [13,14]; the most common are *T. asahii*, *T. inkin, T. faecale* and *T. asteroides* [14–16].

Clinically, *Trichosporon* spp. are associated with superficial and invasive mycoses. These latter can occur as disseminated disease, disease located in major organs and infections that do not affect tissues but are related to medical devices, mainly in urinary and blood infections [4,10,12,14]. Despite its clinical importance, *Trichosporon* spp. are intrinsically resistant to routinely used antifungal agents. Yeasts from this genus are potentially considered multiresistant pathogens because they have low sensitivity to polyenes and azoles. The azole voriconazole is the most appropriate antifungal agent for managing such infections; furthermore, the removal of central venous catheters and recovery from neutropenia are currently recommended for the therapy of patients with invasive trichosporonosis [4,17].

Treatment for patients with trichosporonosis is limited. Many studies use conventional antifungals to test the antifungal susceptibility of *Trichosporon* spp. New possibilities for therapy have been explored, but such studies typically focused on *in vitro* testing and have limited clinical correlation. This scoping review aims to collect information about potential antifungals tested against *Trichosporon* spp.

## Methods

The methods of this scoping review were based on those of the study by Thayabaranathan *et al.* [18].

### Identifying the research question

The research question was as follows: what types of antifungals involving classic drugs, new molecules and biomolecules, and repositioned drugs have been evaluated against *Trichosporon* spp.?

### Identifying relevant studies

Using PubMed and Web of Science, an electronic database search was conducted in groups to find original articles published between 1 January 2010 and 31 May 2020. Two groups were established. The first group was for general search and used the keywords ‘*Trichosporon* and treatment or antifungal’. The second group was a more specific search and used the keywords ‘*Trichosporon’* and ‘treatment or antifungal’ and ‘biofilm’. Only full papers, letters and short communications were considered. All material was published in the English language.

### Study selection

Each article was independently assessed for inclusion and quality. One author performed the initial review of publications using the following inclusion criteria: studies needed to have a focus on *Trichosporon* spp. antifungal susceptibility. In addition, only studies that came from full papers, letters or short communications were considered. The authors separated the articles in groups according to the treatment used (classic antifungals, molecules and biomolecules, repositioned drugs, natural products) using spreadsheets in Google Drive. Characteristics of the articles were recorded in the spreadsheets, such as the article title, author, scientific magazine and publication.

Scientific criteria were set for the exclusion of articles. Articles that did not include *Trichosporon* spp. or did not present clear minimal inhibitory concentration (MIC) results as well as case reports and reviews were excluded. Studies that determined the MIC by guidelines different from the Clinical & Laboratory Standards Institute were also excluded due the scarcity data. The authors filtered abstracts or full articles using the eligibility criteria. When there was uncertainty about inclusion or exclusion, they evaluated the study and made a decision together.

### Article characterization

Articles included in this study were reviewed in detail by all the authors. They were selected to summarize the main data. The following descriptors were used: article aim; classic drugs, molecules and biomolecules, repositioned drugs or natural products tested; study type (*in vitro* or *in vivo*); main results; characterization of clinical isolates of *Trichosporon* spp. (number, species and material in which it was isolated); whether it had been tested on biofilm; protocol and susceptibility method used; and treatment effectiveness determined by MIC determination.

## Results & discussion

### *Trichosporon* spp. isolates

**Figure 1.**
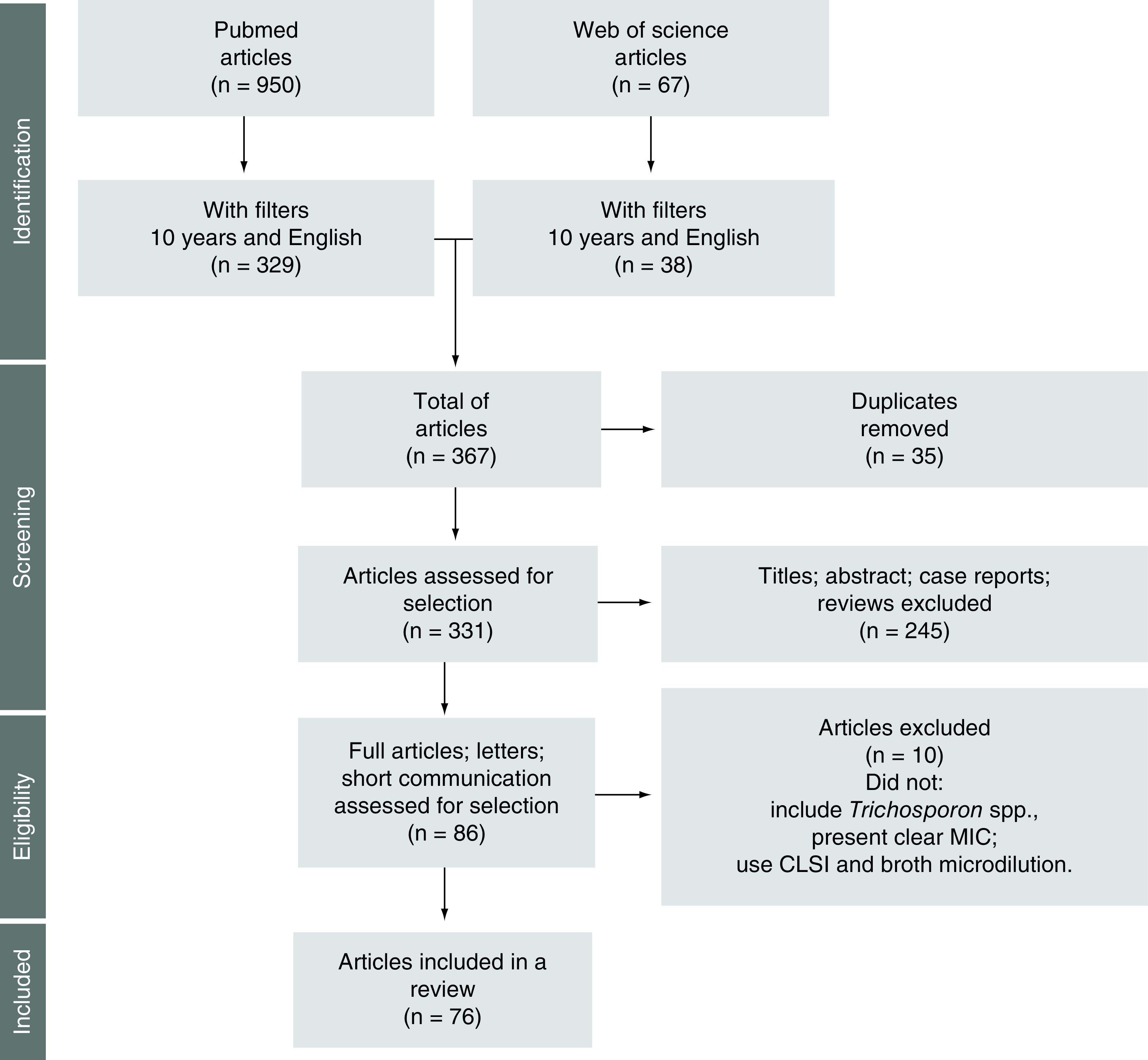
Flowchart for the selection of studies.

Using the previously described method, 76 articles were identified involving several *Trichosporon* spp. (Figure 1); the most studied were *T. asahii*, *T. inkin*, *T. faecale* and *T. asteroides* [14–16]. The majority of clinical isolates were from human biological samples (70 of 76; 92.1%); urine and blood were the most frequent. Fungal infections caused by *Trichosporon* spp. on nails, skin or hair and in fluids, secretions or biopsy materials were also identified. Thus, these fungi do not have a specific human infection site and can be present in any situation according to patient vulnerability.

There were reports (six of 76; 7.9%) of *Trichosporon* spp. in several environmental elements, such as in water and sediments in lakes [19], sewage [20], soil [20] and different animal biological materials [19–25]. For example, *Trichosporon* spp. was identified in the microbiota of captive rheas (*Rhea americana*) [21], gastrointestinal microbiota of chickens (*Gallus domesticus*) [20], ears of dogs and cats with otitis externa [23], different parts (mainly in the walking leg) of red swamp crayfish (*Procambarus clarkii*) [24] and feces of *Agapornis* birds (lovebirds) [25]. Considering these findings, it is important to evaluate possible risks of transmission to humans or animals due to the spread of yeasts via the food chain or environmental routes [22].

### Classic drugs evaluated against *Trichosporon* spp.

Various therapies for *Trichosporon* spp. have been studied over the years. Guidelines have considered that azoles are the primary drug class in therapies, as several species are resistant *in vitro* to amphotericin B (MIC ≥2 μg/ml), flucytosine (MIC 4–128 μg/ml) and echinocandins (MIC >16 μg/ml) [26]. However, different clinical isolates of the same species have great variability in antifungal susceptibility tests [9,10].

In this current scoping review were found 42 articles that evaluated the action of classic antifungals against *Trichosporon* spp. fluconazole (FLC) and amphotericin B (AMB) (31/42; 73.8%) were the most tested antifungals, followed by voriconazole (VRC) (26/42; 61.9%), itraconazole (ITZ) (22/42; 52.3%), caspofungin (CAS) (12/42; 28.6%), posaconazole (POS) (11/42; 26.2%), 5-flucytosine (5-FL) (10/42; 23.8%), micafungin (MCF) (6/42; 14.3%), anidulafungin (ANF) (5/42; 11.9%), terbinafine (TRF) (4/42; 9.5%), isavuconazole (ISZ) (2/42; 4.8%), and finally miconazole (MCZ), ketoconazole (KET), ciclopirox (CIC), amorolfine (AMO) and efinaconazole (EFZ) (1/42; 2.4%), respectively.

**Figure 2.**
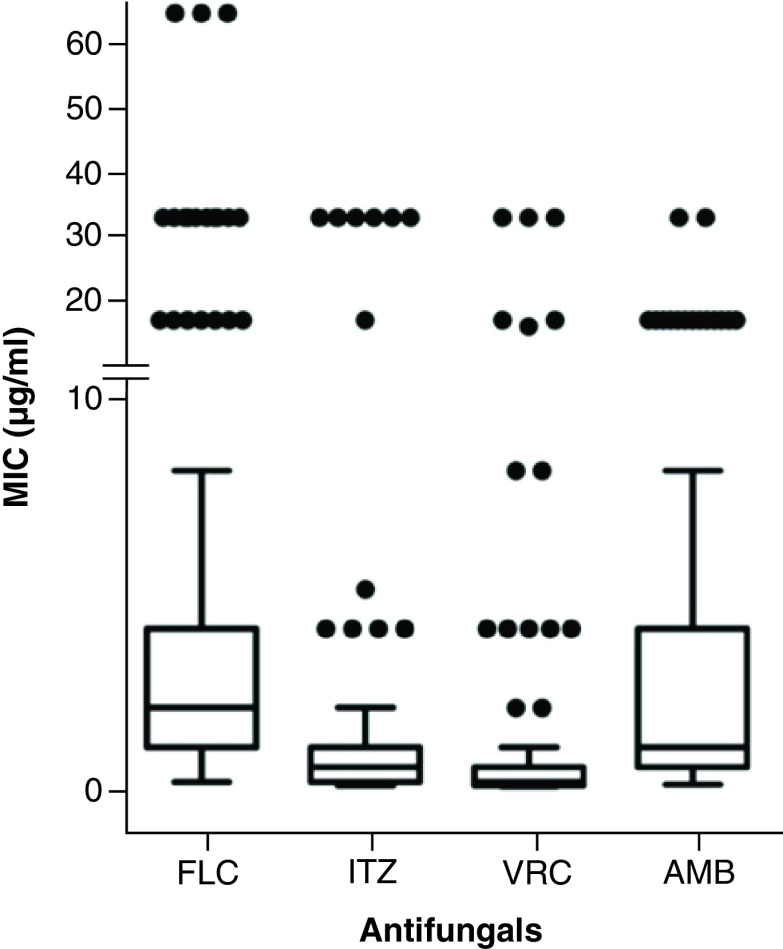
Antifungal susceptibility of FLC, ITZ, VRC and AMB against clinical isolates of *Trichosporon* spp. according to the articles that presented the MIC for each clinical isolate and that used the CLSI protocol from January 2010 to May 2020. The majority of data were compiled in box plot with their standard deviation and those outliers were individually represented (•). AMB: Amphotericin B; CLSI: Clinical & Laboratory Standards Institute; FLC: Fluconazole; ITZ: Itraconazole; MIC: Minimal inhibitory concentrations; VRC: Voriconazole.

There is great variability in the response of this genus to antifungals. Also, comparing different studies is difficult because of the methodologies used, which are often based on the protocols of *Candida* spp. Generally, the observed studies presented results showing MIC variations. In this review, 11 of the observed articles provided individual MIC values for the tested samples (Figure 2) [9–12,27–33]. Although there is this great variability in the MIC of FLC, VRC and AMB, concentrations <10 μg/ml are the most frequently used in antifungal susceptibility tests against *Trichosporon* spp. and were thus used to build the box plot in Figure 2.

Results from several of the observed studies were obtained from the *in vitro* antifungal susceptibility of the fungi in only planktonic cells. However, infections by *Trichosporon* spp. are generally associated with biofilm formation, particularly on invasive medical devices [28]. Interestingly, all articles showed that classic drugs alone do not have a positive effect against *Trichosporon* biofilms [15,22,31,34,35], demonstrating the need for new therapeutic options for this fungal infection.

Regarding *Trichosporon* spp., few studies correlate the *in vitro* susceptibility profile with the clinical response of the patient. Tsai *et al.* [28] reported positive outcomes in patients who provided positive cultures for *T. asahii* and had their clinical characteristics and outcome compared. For five of the patients in this study, MIC values for FLC were low (0.25–2 μg/ml) and they were treated with low doses of FLC (100–400 mg/day); all five were discharged. However, this dosage was not standardized, and more clinical studies are needed to establish guidelines for treatment (e.g., in the management of candidiasis) [36]. Thus, studies similar to those performed by Pfaller *et al.* [37–39] with *Candida* spp. are currently needed for *Trichosporon* spp. because their susceptibility cutoff points are not sufficiently clear to guide patient therapy.

Because of several limitations, such as off-target toxicity and drug resistance, there is a need for new, safe and more effective antifungal agents. In addition, *Trichosporon* spp. often present intrinsic resistance or reduced susceptibility to some drugs, hindering research for potential agents [5].

### Biomolecules & synthetic molecules against *Trichosporon* spp.

**Figure 3.**
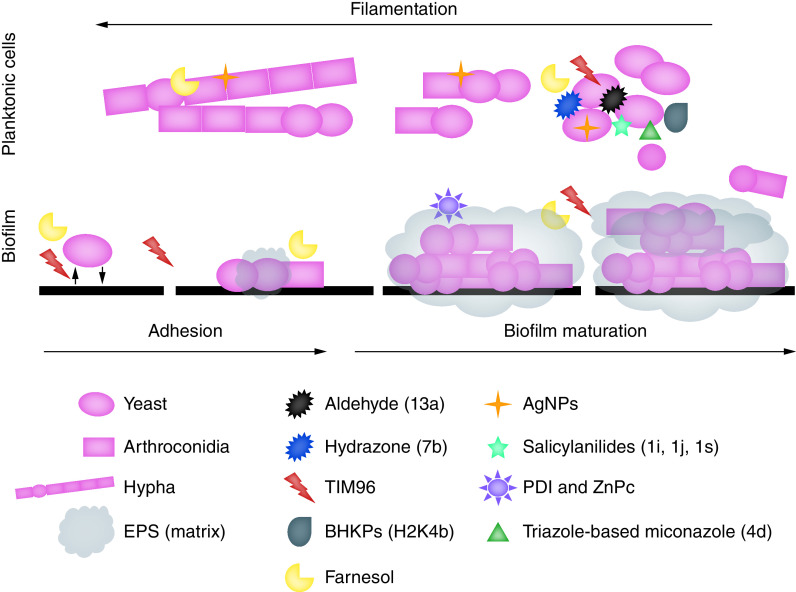
Illustrative image about research of biomolecules and synthetic molecules tested against planktonic and biofilm *Trichosporon* spp. cells from January 2010 to May 2020. Antifungal compound: synthesized aldehydes 4-(2) pyridinyl (13a) and hydrazones 2,3,4-OH (7b). Biosurfactant-producing strain of *Bacillus subtilis* (TIM96). Branched histidine and lysine-rich peptides (BHKPs). Synthesis of silver nanoparticles (AgNPs). Salicylanilide derivatives (1i, 1j, 1s). Cationic nanoemulsion of zinc 2,9,16,23-tetrakis (phenylthio) -29H, 31H-phthalocyanine (ZnPc). 1,4,5-trisubstituted triazole derivatives 1-(1-(2,4-Dichlorophenyl)-2-(1H-imidazol-1-yl)ethyl)-5-pentyl-4-(phenylsulfonyl)-1H-1, 2,3-triazole (4d).

Current research mostly focuses on obtaining or synthesizing molecules that are potentially active against *Trichosporon* spp. and also improving antifungals that are already available [1]. Some studies examined the potential of molecules that act on the *Trischosporon* cell and its virulence factors, such as filamentation, adhesion and biofilm formation (Figure 3). In addition, the syntheses of compounds with specific antifungal action on planktonic and biofilm cells are also being investigated.

Several molecules have been proposed that came from 35 synthesized aldehydes, hydrazones and hydrazines. Examples of these molecules include 4-(2)pyridinyl (13a) and 2,3,4-OH (7b). These molecules have shown promising results against clinical isolates of *T. asahii*. Their MIC values were low (between 8 and 32 μg/ml) with damage to the cell membrane but without ergosterol interaction [40]. The 1-(5-alkyl/arylalkylpyrazin-2-yl)ethylidene]hydrazono}-1,3-thiazolidin-4-ones (11a–11h) obtained from thiosemicarbazones by cyclization with α-chloroacetic acid were synthesized and showed antifungal properties. Several derivatives from this molecule were evaluated, and those with medium length alkyl chains 11a (propyl), 11c (butyl) and 11e (pentyl) were the most potent against *T. asahii* and *Candida* spp., especially 2-{[1-(5-butylpyrazin-2-yl)ethylidene]hydrazono}e-1,3-thiazolidin-4-one (with MIC from 1.14 μg/ml). This suggests antifungal 1,3-thiazolidin-4-ones are versatile compounds; they are synthetic intermediates and potential drugs [41].

Salicylanilides (1a–1t) and their esters with 4-(trifluoromethyl)benzoic acid (2a–2s) as well as and undecylenic acid were tested against different fungal, and 14 salicylanilide derivatives inhibited *T. asahii*. The most antifungal active salicylanilide assayed was N-(4-bromophenyl)-4-chloro-2-hydroxybenzamide (1j). However, the replacement of chlorine by bulkier bromine on the salicylic acid ring and the 4-trifluoromethyl moiety-containing salicylanilides resulted in improved activity [42].

The synthesis of silver nanoparticles (AgNPs) has also been studied for its potential antifungal activity against *Trichosporon* spp. A study reported MIC values of 0.5–1 μg/ml against *T. asahii*. The authors reported mycelium deformation with severe damage on the structure of the cell wall and cell membrane [43]. The potential antifungal properties of histatins were evaluated in tests against fungi with branched histidine and lysine-rich peptides (BHKPs) H2K4b, H3K4b(H) and H3K4B(G) [44]. Differences in susceptibility of BHKPs to fungal species were observed, and *Trichosporon* spp. was most efficiently inhibited by H2K4b.

To improve antifungal activity, studies have used miconazole [45], 1,4,5-trisubstituted derivatives [46] and novel oxazolidin-2-one-linked 1,2,3-triazole derivatives [47]. Against *T. cutaneum*, the greatest reductions were with the antifungal compound 1-(1-(2,4-dichlorophenyl)-2-(1H-imidazol-1-yl)ethyl)-5-pentyl-4-(phenylsulfonyl)-1H-1,2,3-triazole (4d) with a MIC of 0.12 μg/ml [46]. The authors observed that an alkyl group in 5-substituted triazole likely improves interaction with the 14-a-demethylase (P45014DM, CYP51) enzyme, leading to its selective inhibition and the inhibition of fungal cell growth [46].

Antifungal activity on *Trichosporon* biofilm was reported from the use of 10× MIC of a *Bacillus subtilis* (TIM96) biosurfactant-producing strain that reduced cell adhesion by interfering in biofilm formation. TIM96 reduced the cellular ergosterol content, altered the membrane permeability and the surface hydrophobicity [48]. Another study examined the inhibitory effect of farnesol against planktonic and biofilm cells of clinical *T. asahii* and *T. inkin*. Farnesol inhibited planktonic growth, filamentation, adhesion and biofilm development, demonstrating its potential as an antibiofilm molecule [49]. The potential of a cationic nanoemulsion of zinc 2,9,16,23-tetrakis(phenylthio)-29H,31H-phthalocyanine (ZnPc) has also been reported because its use with photodynamic inactivation caused a reduction of 0.85 log for biofilms formed by *T. mucoides* [50].

The similarities between fungal and mammalian cells impede the development of novel antifungals with ideal characteristics, such as broad-spectrum effectiveness, enhanced bioavailability, minimal toxicity and minimal side effects. Consequently, drug development against invasive fungal pathogens has been slow.

### Drug repositioning as an alternative to new therapeutic options

Drug repositioning is an alternative strategy to new antifungal therapeutic options against *Trichosporon* spp. Previously approved compounds used for other purposes or drugs that were shelved after failure in clinical trials could be quickly and inexpensively brought into clinical practice [51,52]. Some previously approved compounds have been evaluated regarding antifungal properties.

Sertraline, an antidepressant and anxiolytic from selective serotonin reuptake inhibitor class, exhibited synergic effects *in vitro* against *T. asahii* with AMB, CAS and FLC in planktonic cells and with AMB in biofilm forms [53]. The antifungal activity of this antidepressant was first observed in three patients with premenstrual dysphoric disorder and recurrent vulvovaginal candidiasis (CVVR). Clinical symptoms of CVVR disappeared in these patients after treatment with sertraline during therapy. However, they returned when the treatment was interrupted [54]. Other *in vitro* studies with sertraline showed its antifungal activity against *Aspergillus* [55], *Candida* [56,57] and *Cryptococcus* [58,59]. Sertraline has also shown *in vivo* anticryptococcal activity [60], but randomized clinical studies for cryptococcal meningitis treatment with sertraline have not shown positive results [61,62].

Tacrolimus is an immunosuppressant widely used for the prevention of transplant rejection, blocking the immune system through inhibition of calcineurin. It was tested against *T. asahii*. *In vitro* results showed a synergic effect with AMB and CAS on azole-sensitive isolates but not against resistant strains [63].

Nonsteroidal antiinflammatory drugs, such as aspirin, ibuprofen and diclofenac sodium, have shown potential antifungal activity against pathogenic fungi under planktonic and biofilm forms [64]. One study indicated the potential effects of AMB/ibuprofen and CAS/ibuprofen combinations against *T. asahii* isolated from patients with trichosporonosis [65].

Ritonavir, an HIV aspartyl protease inhibitor, was tested against *T. asahii* and *T. inkin*. This drug inhibited *Trichosporon* growth, reduced protease activity, decreased cell adhesion and biofilm formation and also altered the structure and matrix composition of the biofilm. Synergism was not observed between ritonavir and antifungals [66].

Diphenyl diselenide and ebselen, which mimic the antioxidant effect of the glutathione peroxidase, were tested against *Trichosporon* strains. Both compounds showed synergism with some antifungals, mainly with AMB and some other tested antifungals. However, they demonstrated antagonism when combined with FLC [67].

Sodium butyrate, a histone deacetylase inhibitor, reduced the adhesion, biofilm metabolic activity and biomass of *T. asahii* and *T. inkin* [68]. In addition, this compound inhibited planktonic growth, interfered with the filamentation of these species, affecting adhesion, development and maturation of the biofilm.

Although drug repositioning is an interesting alternative, it could be applied more appropriately to finding efficient antifungal activity addressing *Trichoporon* spp. The selection of compounds for experimental investigation should be based on in-depth studies on specific fungal targets, such as the use of high-throughput screening that integrates automation and computational advances with biological knowledge. This allows for evaluation of thousands or even millions of molecules and increases the chances of finding drug candidates [51]. High-throughput screening can be performed using *in silico* methods [69] or *in vitro* experiments [70]. Comparative genomics should also be used when selecting novel compounds, which allows for safe molecule targeting when acting against certain fungal targets [71].

### Antifungal studies of natural products

Natural products have historically been a source of antifungal drugs, such as the polyenes nystatin and AMB, which are potent antifungal antibiotics against a large number of fungi and were initially isolated from strains of *Streptomycetes* [72]. In the context of the current review, we found 15 published articles involving natural products evaluated against species from the *Trichosporon* genus. Details regarding 13 of these articles are shown in Table 1. However, the likelihood of discovery of new natural products for antifungal treatments is remote.

**Table 1.** List of anti-*Trichosporon* natural products reviewed considering origins, antifungal properties and main experimental results.

Compound	Major components	Source	*Trichosporon*		Antifungal test			Ref.
			Species	Strains origin	Method	Measure	Results	
Regrapex-R-forte^™^	Resveratrol	*Vitis vinifera Polygonum cuspidatum*	*T. cutaneum*	CCY 30-5–10	Broth microdilution	MIC	0–222 μg/ml^†^	[80]
Essential oil	α-pinene	*Rosmarinus officinalis*	*Trichosporon* sp.	Dogs and cats	Broth microdilution	MIC	0.46 μg/μl	[23]
Berberine hydrochloride	Berberine	Synthesized	*T. asahii*	Human	Broth microdilution	MIC	32–128 μg/ml	[81]
Essential oil	Isocaryophillene	*Psidium cattleianum*	*T. asahi*	NA	Broth microdilution	MIC	41.67 μg/ml	[73]
Essential oils	NI	*Kunzea ericoides* *Leptospermum scoparium*	*T. mucoides*	ATCC 204094	Broth microdilution	^‡^	0.78 μg/μl 1.56 μg/μl	[74]
								
Ozonized sunflower oil	Bioperoxoil^®^	Synthesized	*T. asahii*	Human (n = 10)	Agar disk diffusion	Inhibition zone	19 mm	[82]
Essential oils	Eugenol, geraniol and others	25 aromatic plants	*T. ovoides*	NCYC 2796	Agar well diffusion	MFC	1.55–400 μl/ml	[75]
Native venom	Crotamine	*Crotalus durissus terrificus*	*Trichosporon* sp.	IOC 4569	Broth microdilution	MIC	12.5– 25.0 μl/ml	[83]
Ethanolic extract	Honokiol magnolol	*Magnolia dealbata*	*T. beigelii*	Human (n = 1)	Disk diffusion	Inhibition zone	11 mm	[76]
Essential oil	Linalool	*Homalomena aromatica*	*T. beigelii*	NCIM 3326	Agar well diffusion	MIC	10 μg/ml	[77]
Hydroalcoholic extract	Propolis	Honeybee	*Trichosporon* sp.	Human (n = 1)	Broth microdilution	MIC	0.0125 μg/ml	[79]
Four extract kinds^§^	NI	*Solanum melongena*	*T. beigelii*	NA	Agar diffusion	Inhibition zones	27.0–35.5 mm	[78]
Essential oil	Phenylheptatriyne	*Bidens cernua*	*T. cutaneum*	Various (n = 5)	Broth macrodilution	MFC	200 μg/ml	[20]

† If diluted in 40% EtOH and DMSO.

‡ The measured values (% v/v solution).

§ Extracts (petroleum ether, chloroform, methanol and water).

MFC: Minimal fungicidal concentration; MIC: Minimal inhibitory concentration; NA: Not available.

A variety of natural products have been explored for their antifungal activity, and most come from plants [20,23,73–78]. Studies have examined the antifungal activity of compounds produced by bees (propolis) [79], synthesized from natural products [80–82] and extracted from snake venom [83]. However, no promising candidates for drugs from natural products were found. As far as we know, no *in vivo* studies have been performed with natural products that targeted *Trichosporon* spp., including in experimental animals.

*In vitro* tests of antifungal activity from natural products have been used in only a few small studies. One study tested a compound *in vitro* against a *Trichosporon* spp. biofilm [80]. According these authors, resveratrol and Regrapex-R-forte (a dietary supplement that contains the extract of *Vitis vinifera* grape and extract of *Polygonum cuspidatum* root) showed antibiofilm effects. They both inhibited biofilm formation and eradicated mature biofilm. The *in vitro* results with this compound were comparable to AMB, the most efficient antimycotic agent [72]. Such antifungal action could be added to a nutritional effect, recomposing the endogenous intestinal microbiota, and thereby avoiding the dysbiosis, a relevant point today [84].

However, the use of various methods to assess the antifungal properties of natural products hampers study comparison. In addition, several studies only reported data from agar disk diffusion, which provides information from a simple screening. The MIC and minimal fungicidal concentration (MFC) provided by dilution tests are more contributory and allow for the observance of greater variability. In general, MIC values ranged from 0 to 200 μg/ml among the compounds studied. All of them showed significant *in vitro* activity according to preestablished criteria [85], as MIC values were between 100 and 625 μg/ml. Despite these promising preliminary results, these compounds have a long road ahead before they will be eligible for clinical trials. Some of these compounds should be evaluated for *in vitro* and *in vivo* toxicity and their pharmacological potential.

## Conclusion & future perspective

In the past 10 years, new potential antifungals for trichosporonosis treatment have not been found, and the need for effective drugs remains. Several compounds have been tested, but none have shown promising results. In general, most studies did not present appropriate scientific criteria for drug tests, compromising their quality.

Thus, new studies presenting well-defined scientific criteria are essential. For example, the selection of compounds for experimental investigation needs to be suitable and based on in-depth studies on fungal cell targets. In addition, new studies need to address *Trichosporon* biofilms (an important drug-resistance factor) and correlate susceptibility *in vitro* with patient clinical response. Another strategy would be to pursue further tests on *in vitro* and *in vivo* toxicity of drugs that are already under study and also evaluate their pharmacological potential.

**Executive summary**

**Background**

- Trichosporonosis has been increasing worldwide, and there are no effective treatments for it.

**Aim**

- To collect information about potential antifungals tested against *Trichosporon* spp.

**Methods**

- A search in PubMed and Web of Science for relevant articles regarding potential antifungals for *Trichosporon* spp.

**Results & discussion**

- Related articles were divided into three topics according to the origin of the compound, such as biomolecules and molecules, repositioned drugs and natural products.

**Conclusion**

- Several compounds have been tested but without promising results.
- Most studies did not present appropriate scientific criteria for drug tests thus compromising the quality of the research.
